# Evaluation of rock mass units using a non-invasive geophysical approach

**DOI:** 10.1038/s41598-023-41570-y

**Published:** 2023-09-03

**Authors:** Muhammad Hasan, Yanjun Shang, Qingsen Meng

**Affiliations:** 1 Key Laboratory of Shale Gas and Geoengineering, Institute of Geology and Geophysics, Chinese Academy of Sciences, No. 19, Beitucheng Western Road, Chaoyang District, Beijing, 100029 People’s Republic of China; 2 College of Earth and Planetary Sciences, University of Chinese Academy of Sciences, Beijing, 100049 People’s Republic of China; 3 Innovation Academy for Earth Science, Chinese Academy of Sciences, Beijing, 100029 People’s Republic of China

**Keywords:** Environmental sciences, Hydrology, Solid Earth sciences, Engineering

## Abstract

Thorough and accurate assessment of rock mass units is important for development of engineering infrastructures and groundwater resources assessments. Rock mass units are widely evaluated by reliable geomechanical parameters namely rock quality designation (RQD) and rock core index (RCI). Conventionally, these parameters are acquired via an extensive number of geotechnical tests. Such tests, however, suffer efficiency for data coverage, cost, equipment and topographic constrictions, and hence cause ambiguity in geological models for a detailed evaluation of rock mass integrity. Conversely, geophysical surveys offer fast, more user-friendly, less invasive, more cost-effective and less time-consuming approach for geological investigations. The past research confirms a useful link between geophysical and geotechnical parameters. But, none of the past studies provides a suitable and generalized relation between these parameters which can reduce geotechnical model uncertainty mostly caused by inadequate data and subsurface heterogeneity. This paper proposes a meaningful and feasible method to obtain geomechanical parameters using a certain number of drillings and geophysical data of four different sites. Based on electrical resistivity obtained from electrical resistivity tomography (ERT) and controlled-source audio-frequency magneto telluric (CSAMT), this research provides the general and adaptable formulas for geotechnical parameter estimation and reduces geological model uncertainty for more detailed 2D/3D imaging of RQD and RCI covering the whole sites where even no drilling data exists. Thus, the investigated sites are assessed laterally and vertically along each geophysical profile via distinct value ranges of geological parameters for a thorough and reliable evaluation of rock mass units in highly heterogeneous setting. Our research reduces the ambiguity caused by structural heterogeneities and scarce data, fills the gap between inadequate well tests and the true geological models, and gives new insights into the rock mass units for proper engineering design and groundwater exploitation.

## Introduction

Accurate assessment of geological models for engineering design and groundwater evaluation is extremely challenging in geotechnical engineering^1,2^. Inadequate characterization of subsurface rock units often leads to structural dilapidation and potential foundation-related failures^3–5^. Given the natural heterogeneity and spatial variability in the subsurface environments, detailed geological knowledge of a construction site is necessary in order to propose structural foundations and effective earthworks^1,6–9^. The necessary bearing competency for engineered structures is provided by the foundation rocks^10,11^. Geomechanical parameters are mainly used to estimate the rock mass bearing potency in engineering construction and groundwater exploitation^12–15^.

Rock mass evaluation is mostly carried out using geomechanical parameters, namely rock core index (RCI), Young’s modulus (E), rock quality designation (RQD), volumetric joint counts (Jv), and rock mass integrity coefficient (Kv)^1,2,10, 15–19^. RCI and RQD provide basis for understanding the rock characteristics. These parameters divide the integrity of rock mass into different ratings to determine the load-bearing and water-bearing reliability^20,21^. Traditionally, RCI and RQD are acquired from rock cores of borehole tests^12,15–17,22^. However, it is always a difficult task to obtain RQD and RCI from the numerous drilling tests, since it is never easy to accurately locate place of collection samples (even up to 200 m) which in addition are so diverse in terms of the degree of fracture. Such techniques offer only point-scale vertical measurements, are difficult to carry out in steep topographic terrains and need more equipment^3,10,17^. Moreover, geotechnical tests are high-priced and time-consuming^16,21^. Hence, drilling tests can barely meet the engineers’ necessities^20^. Thus, the inadequate geological knowledge obtained from these tests cause ambiguity in the geological models of foundation rocks. Therefore, a –non-invasive geophysical approach to fill gap between reliable models and insufficient drilling data is indispensable for completion of engineering structures and groundwater resources appraisal.

Many geophysical methods, namely electrical resistivity tomography (ERT), ground penetrating radar (GPR), induced polarization (IP), controlled source audio frequency magnetotelluric (CSAMT), seismic refraction tomography (SRT), self-potential (SP), magnetic and gravity are widely carried out in geotechnical, environmental and groundwater studies^1,6,15,17,18,23–37^. Compared to the borehole approaches, geophysical methods offer volumetric measurements of the subsurface^28,38^. Furthermore, such methods are non-destructive, non-invasive, relatively faster, economical and comparatively user-friendly^25,39^. Both geomechanical and geophysical parameters are controlled by subsurface heterogeneities. Many authors have successfully linked geophysical parameters with geomechanical parameters^1,12–16,18,20–22, 40–46^. However, such correlation is a challenging task, since these parameters depend on rock type, subsurface lithology, weathering intensity/degree, fractures and faults, water content, rock deformation, pores connectivity, permeability, rock alteration, and rock association^1,12,19,20^. Recently, several investigations were carried out using different geophysical methods for predicting the spatial distribution of geotechnical parameters and transforming the geophysical information into geotechnical information that engineers can understand more easily. Junaid et al.^47^ integrated ERT with RQD and Jv for rock mass quality evaluation in the north–south expressway (NSE) of Malaysia. Hasan et al.^1^ estimated RQD of tuff rocks using ERT measurements in Huizhou (China). Alemdag et al.^48^ determined RQD in metamorphic rocks of Bayburt-Kırklartepe Dam (Turkey) using P wave velocity (V_p_) of SRT method. Johora et al.^49^ used artificial neural networks (ANNs) to predict geotechnical parameters from seismic wave velocity. Vagnon et al.^50^ combined seismic and electrical resistivity data to derive geotechnical parameters in Piedmont Region (Italy). Hasan et al.^15^ linked electrical resistivity with Kv for site suitability of engineering infrastructure in Guangdong (China). Sousa and Gomes^14^ obtained geotechnical parameters via correlation between the Cone Penetration Test (CPT) tests and electrical resistivity survey in tailings dams (Brazil). Salaamah et al.^22^ estimated RQD value of volcanic rocks from the P-wave velocity via linear regression analysis. Zhang et al.^21^ integrated electrical resistivity with the basic engineering parameters to provide assistance to engineering design in Jiangsu (China). Lin et al.^16^ incorporated seismic velocity with RCI for rock mass characterization of shallow granite. Moreover, geotechnical parameters can also be predicted from geophysical methods using statistical fuzzy approaches without the need of any predefined equation between the measured geophysical and the predicted geotechnical approach^51,52^.

Different geophysical methods can contribute in a different way for the investigation of the subsurface; in general, an integrated geophysical approach can be the most suitable way to reduce the uncertainty in the interpretation of geophysical models. Many researchers have used an integrated study of more than one geophysical method in their geotechnical studies to reduce the geological uncertainty caused by subsurface heterogeneities and insufficient data. Hasan et al.^53^ performed an incorporated investigation of four geophysical methods (ERT, IP, SP and magnetic) methods coupled with RQD to investigate a hard rock site for successful installation of Accelerator Driven System (ADS) in South China. Ganguli et al.^54^ combined magnetic and gravity data to reduce ambiguity in the evaluation of crustal architecture across Salem Attur Shear Zone (India). Srivastava et al.^55^ carried out a time-lapse study of ERT and SP methods for old mine assessment in Raniganj Coalfield (India). L´evy et al.^56^ performed field investigations of geothermal areas in Krafla (Iceland) using an incorporated approach of ERT and time-domain IP. Wang et al.^57^ obtained the resulted models with better resolution via joint inversion of ERT and radio-magnetotelluric (RMT) data in a hard rock laboratory site (Sweden). Drahor and Berge^58^ carried out an integrated investigation of six geophysical methods (ERT, SRT, GPR, SP, magnetic and VLF (Very Low Frequency electromagnetic)) for faulting characteristics in Western Anatolia (Turkey). Voytek et al.^59^ used the combined ERT and SP methods to evaluate hydrologic flowpaths on arctic hillslopes in Alaska (USA). Samyn et al.^60^ investigated the sinkhole hazard in Orleans (France) using an incorporated study of seismic and electrical resistivity methods. Marescot et al.^61^ solved the uncertainty in water-clay evaluation for slope instability investigation by combining ERT with IP in the Swiss Alps. ERT is progressively being used in geotechnical engineering to achieve 2D/3D detailed mapping of the subsurface^1,15,18,27,28, 32^. However, ERT can only assess the subsurface at shallow depths^32,62^. CSAMT, on the other hand, is becoming the most suitable and economical geophysical method to obtain detailed information of the subsurface at large depths^17,31,63–66^. Resistivity is a function of rock porosity and, consequently, the degree of fracturing in the rock mass; it is also strongly controlled by other factors including rock type, mineral and water contents, joint fissures development, temperature, pressure and pore-water electrical conductivity (EC). Such factors may vary substantially from one geotechnical site to another or even within a site. Based on above factors, empirical equation obtained from a specific site using resistivity of single geophysical method may cause ambiguity in geological model for the estimation of geomechanical parameters. Therefore, to reduce uncertainty in geological model, empirical approach based on electrical resistivity of different geophysical methods (ERT and CSAMT) obtained from different sites is the need of time to propose a generalized correlation for more reliable estimation of important geomechanical parameters (RQD and RCI).

**Figure 1:**
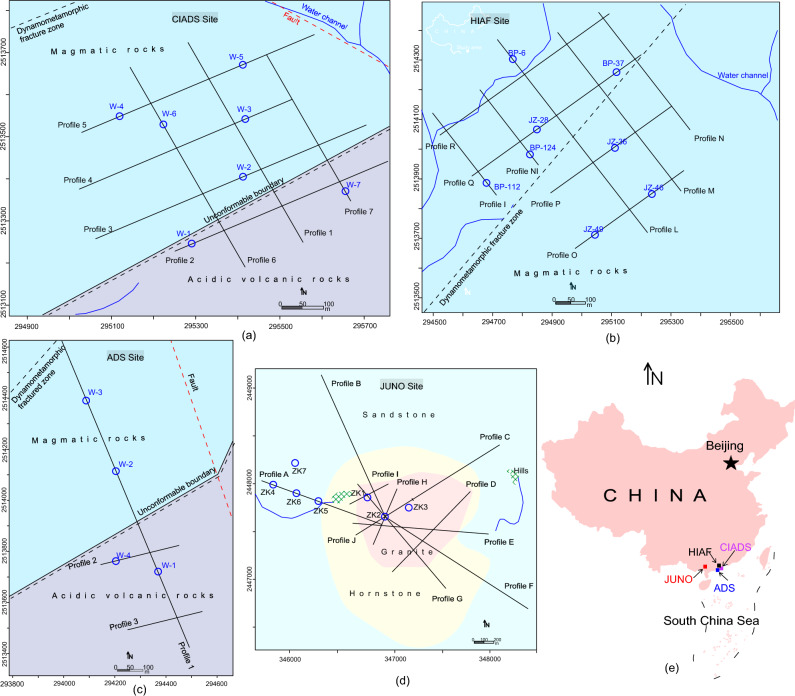
Location of the project sites with the main geological settings (regions of different colors), geophysical profiles (black lines), boreholes (blue circles), main faults (dotted red lines), an unconformable boundary (black line with the dotted line), dynamometamorphic fractured zone (black dashed line), water channels (blue lines), hills (green yellow region), including (**a**) CIADS site with 7 ERT profiles and 7 drill tests, (**b**) HIAF site with 9 ERT lines and 8 boreholes, (**c**) ADS site with 3 ERT profiles and 4 boreholes, (**d**) JUNO site with 10 CSAMT profiles and 7 drill holes, and (**e**) China map with all sites located in South China.

In the proposed approach, we suggest to carry out few boreholes only at some important locations with suitable depth covering all types of rocks (ranging from low to high resistive rock). Then more reliable electrical resistivity surveys such as CSAMT and ERT can be conducted to evaluate the whole project area. Afterwards, a correlation between drilling and resistivity data would be performed to acquire geomechanical parameters in the whole study site where even no borehole is found. In this contribution, we used integrated data of ERT and CSAMT surveys coupled with limited boreholes from four different sites (Fig. 1) to obtain a general and adaptable correlation between electrical resistivity and RQD/RCI. The obtained empirical formulas were used to generate a detailed 2D/3D imaging of RQD and RCI at one site (Fig. 1a). The correlation of RQD and RCI with resistivity obtained in this research is important and contributes in the promotion of geophysical methods as an addition tool in geotechnical studies. The empirical equations derived from four different sites for the prediction of RQD and RCI can be useful for many researchers in areas where borehole data is not available.

Our proposed research, compared with the existing studies, provides the general and adaptable equations for both shallow and large depth imaging of important geomechanical parameters (RCI and RQD) using geophysical and borehole data of four different sites. This is the only work which uses CSAMT to obtain geomechanical parameters. This research provides correlation between electrical resistivity obtained from two geophysical methods (ERT and CSAMT) and RCI/RQD determined from the limited borehole data to obtain more reliable geotechnical parameters. Besides, an important geomechanical parameter RCI has not yet been determined using any geophysical method. The main objectives of this work are: (1) to introduce geophysical methods as an integral part of a geotechnical study; (2) to reduce geological model uncertainty caused by limited data and subsurface heterogeneities, and fills the gap between true geological models and scarce data; (3) to reduce a large number of costly drills for a detailed mapping of rock mass units; (4) to provide general equations for the estimation of geomechanical parameters using electrical resistivity; and (5) to obtain more detailed and accurate imaging of RQD/RCI for both shallow and large depth in different geological settings..

## Study areas and hydrogeological setting

The present research was employed by geophysical and borehole data obtained from four different sites of China, namely CIADS (China Initiative Accelerator Driven System) (Fig. 1a), HIAF (High Intensity Accelerator Facility) (Fig. 1b), ADS (Accelerator Driven System) (Fig. 1c) and JUNO or JNEB (Jiangmen Neutrino Experimental Base) (Fig. 1d). The ADS and HIAF sites are situated in South Huizhou, while CIADS site is situated in Dakengkou, Huidong County, coastal area of South China Sea, and the JUNO site is located in Jiangmen city, Guangdong (Fig. 1e). The detailed investigation of CIADS site is provided in this work.

The CIADS, HIAF and ADS sites (Fig. 1a–c) are surrounded by the lush green hills of tuff rocks. The coastal side is mostly dominated by Quaternary strata. The project areas are mainly exposed by the Jurassic rocks such as magmatic and acidic volcanic rocks. Tuff rock is dominated with various types of rocks, including highly integral, relatively integral, poorly weathered/fractured, relatively weathered/fractured and highly weathered rock. In the recent past, there occurred no volcanic activity. The magmatic action is distinguished by metamorphism, tectonics and geological periods. The South China Fold System is the main stratigraphic unit of Huidong County. The Piyang-Zijin fold, Yanshanian movement, Shuangyunshan-Lianhuashan uplift block, Huiyang-Yongmei depression, Yunshan Mountain and coastal fault block formed the regional geological setting of Huidong County including fractures, faults, folds, unconformable boundaries, dynamo metamorphic zone, anticline and syncline structures, and torsional, shearing and tensional structures. Since 1100, over 35 earthquakes with magnitude 5–7 were recorded. The project areas are situated in the Monsoon (rainy season) Region^67^. Huidong County records annual average rainfall of 1950 mm, including number of typhoons, cyclones and tornadoes every year. South China has plentiful geothermal resources of hot water. Groundwater in the weathered/fractured layer is dynamically formed by the weathering, tectonic and hydrothermal processes^67^.

The JUNO site (Fig. 1d) belongs to the tropical Monsoon climate zone of Southeast Asia with strong typhoons coming during summer and autumn. The annual average temperature is 22 °C, the humidity is 82%, and the annual rainfall is 2300 mm. Geomorphologically, the project site is a moderately low mountainous area, generally high in the south and low in the north with topography of 20–500 m and terrain slope of 10°–20°. In the northeast of the site, there is a small river in Yongkouwei. The subsurface vegetation is well developed with dense vegetation but poor field working condition. The engineering site mainly exposes Quaternary, Ordovician, Cambrian, Devonian, Carboniferous, Permian, Jurassic, and Paleogene strata, including Caledonian, Indosinian and Yanshanian intrusive rocks. The dominant lihologies include sandstone, hornstone, highly weathered/fractured granite, poorly weathered/fractured granite and fresh granite^67^. Geological structure of the site area belongs to the Kaiping concave fold fault. Due to the influence of multiple structures and magmatic activity, the geological structure of this area is relatively complicated. The main fault strikes in the site area are basically consistent with the regional tectonic line, and the development of joint fissures reflects the background of multiple geological periods of tectonic movement in this area.

## Methods

**Figure 2:**
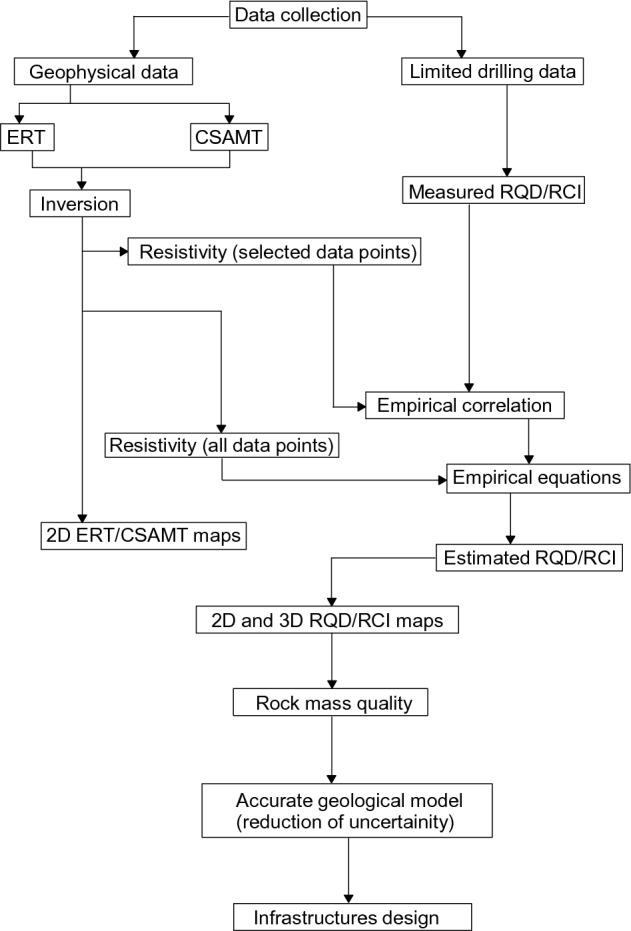
A flowchart of our work including the important methodology steps to obtain a reliable geological model for infrastructure development.

The present work was carried out using an incorporated geophysical approach of ERT and CSAMT coupled with inadequate boreholes in order to obtain important engineering parameters (RQD and RCI) for large coverage of area. The key steps of the proposed research are given in a flowchart (Fig. 2).

### ERT survey

ERT is a commonly used geophysical practice that can investigate the heterogeneous subsurface with high-resolution lateral and vertical imaging of resistivity^1,17,18,27,28,32, 39^. Electrical resistivity measured in ERT surveys is controlled by different factors, including subsurface rock/lithology type, mineralogy, weathering degree, clay, fractures and faults, porosity, rock association, temperature, water content, pores electrical connectivity, salinity, permeability and rock alteration^15,32^. Igneous and metamorphic rocks have the highest resistivity value (up to 10^8^ Ωm)^62^. Volcanic tuff rock is igneous rock, which has resistivity value of 10–10^3^ Ωm for saturated weathered/fractured rock and 10^3^–10^6^ Ωm for fresh rock^1^. In this survey, the highest resistivity of volcanic rock was obtained up to 10^5^ Ωm. In ERT, an extensive number of electrode measurements are used to obtain 2D and even 3D models of electrical resistivity^62^. ERT uses electrical current that is transmitted into the geological layers using two outer current-electrodes, while the difference in electrical voltage is computed via two inner potential-electrodes. ERT can be performed using various electrode combinations, i.e., pole-pole, Wenner, pole-dipole, Schlumberger and dipole–dipole. Each array features various advantages and disadvantages in terms of field set-up, lateral resolution, signal strength and investigation depth or vertical penetration, but generally all follow the same general rules i.e., the larger the electrode spacing and profile length, the deeper the penetration/investigation depth (this may not be true for dipole–dipole and pole-dipole under different conditions)^62^. Where, ERT profile length is obtained by the product of electrode interval and total electrodes. The investigation depth for pole-pole array is 0.9 times of the total ERT spread; however, the investigation depth for most of other arrays is 0.2 times of the total surveyed length^68^. The Wenner array is more suitable in a noisy area for good vertical resolution, the dipole–dipole array is an attractive choice for good horizontal resolution and data coverage, the Wenner-Schlumberger array is useful for both good vertical and horizontal resolution, the pole-pole array with small electrode spacing is a reasonable choice when deepest investigation depth and widest horizontal coverage is required, and the pole-dipole can be useful to evaluate vertical structures with good horizontal coverage using limited electrodes in both forward and reverse measurements^62^. The uncertainty caused by a single array can be reduced by applying two or more arrays^62^.

**Figure 3:**
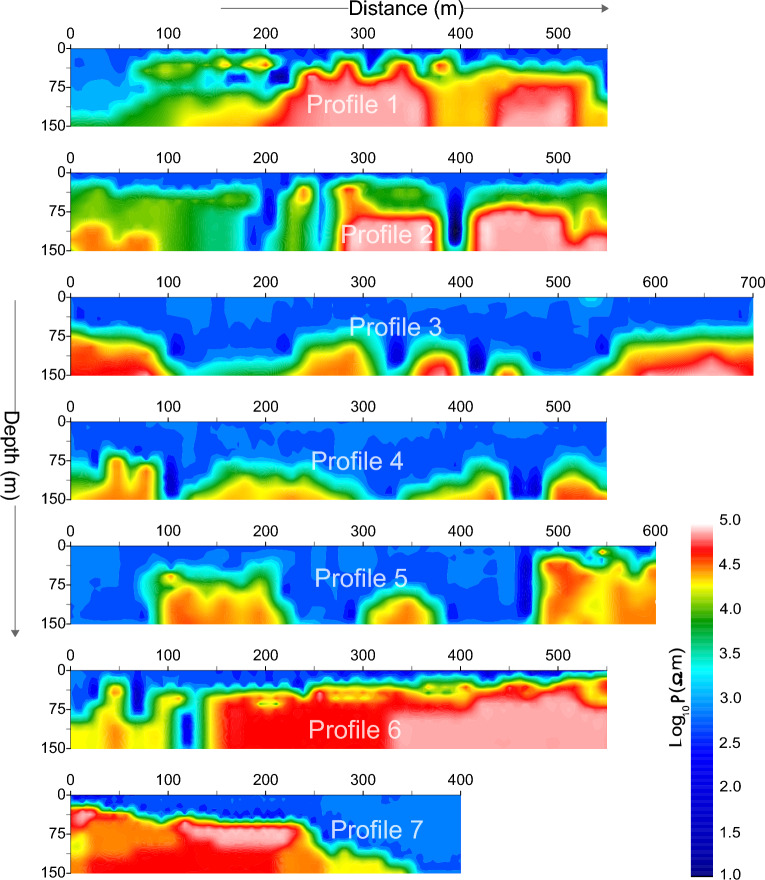
The obtained 2D ERT models (with resistivity increasing from dark blue to red white on a color-bar) along 7 geophysical profiles 1–7 in CIADS site, including the investigation depth of 150 m and profile distance of 400–700 m.

In ERT survey, apparent resistivity measurements were attained by a multi-electrode Imaging Terrameter SAS 4000 (ABEM, Inc.) of 48 electrodes, including multi-electrode convertor, multi-functional electric instruments, multi-core cables and a Syscal Junior resistivity meter. ERT survey was carried out for 150 m depth (depth of investigation) in CIADS, 200 m depth in HIAF and 100 m depth in ADS site. We used pole-pole to acquire ERT data because widest horizontal coverage and deepest investigation depth were desired from this survey. However, compared with other arrays, its resolution is poor. In this study, the target zones are mainly at shallow depth since the fresh rock is dominant below few meter depth. The large distance between the pole-pole potential electrodes causes telluric (random) noise which was removed by the inversion technique. The pole-dipole uses only one electrode (current electrode) at infinity distance; however, pole-pole uses a current electrode opposite to a potential electrode at infinity distance. Therefore, compared with pole-dipole and dipole–dipole, pole-pole uses more space, which makes it less common than other arrays^69^. However, there was no such issue of space in the project sites to use pole-pole. The surveyed lines were expanded using Roll-Along mode. ERT survey with electrode-interval of 5 m was conducted in three sites namely CIADS, HIAF and ADS, such as CIADS with 7 geophysical profiles 1–7, HIAF with 9 ERT lines 1–9 and ADS with 3 geophysical profiles 1–3. The data quality was ensured using maximum 10 stacking^62^. Electrical resistivity data was processed by RMS (root-mean-squares) error under 5%. For ERT data processing, the computer code of RES2DINV was performed to invert the observed apparent resistivity data sets^70^. This software generates the 2D resistivity pseudo-section using a non-linear optimization technique^71^. In this study, the L_2_ norm optimization method or least squares smoothness-constrain^72^ (which can minimize the square of the changes in the model resistivities) was chosen because this inversion method is very useful when resistivity is changing smoothly in the subsurface from fractured zones to more competent zones^68^. Therefore, such inversion is more suitable in the investigated sites where we have a smooth transition of completely weathered/fractured rock into poorly weathered/fractured rock and partly fractured rock into fresh rock. The L_1_ norm optimization method or robust modeling constraint (which tries to minimize the absolute changes in resistivities) is useful when subsurface resistivity is changing sharply (i.e., bedrock-soil interface)^62^. True resistivity should not exceed 20 times the maximum apparent resistivity^69^; we obtained the highest inverted resistivity value less than 100,000 Ωm for maximum apparent resistivity value of 4500 Ωm. All models were generated after 8 iterations. The model sensitivity is an estimate of the knowledge related to the model-block resistivity contained in the measured data set^68^. The model reliability mainly depends on the model sensitivity. (i.e., high sensitivity provides more accurate models). Overall, the model sensitivity is higher near the surface (near electrodes). Generally, model sensitivity decreases significantly towards the bottom of the ERT plane, revealing considerable loss of resolution in this part^62^. The model resolution increases with model sensitivity. By using different settings of inversion parameters in RES2DINV based on geological conditions of the investigated sites and nature of data sets, the inversion model uncertainty was reduced and the reliable/acceptable inversion models were obtained. The ERT models obtained from CIADS site are shown in (Fig. 3).

### CSAMT survey

CSAMT is a hybrid method progressively being used in geotechnical engineering^17,31,63–66^. It measures subsurface resistivity distribution of the earth’s electric and magnetic fields, mainly due to the time-dependent variations and the high-frequency electromagnetic waves artificially transmitted by non-polarization. In CSAMT, the material’s resistivity is computed by magnetometers and electric dipoles in two directions. The CSAMT principle is based on the complete unified electromagnetic field theory of Maxwell's equations. Using the natural field source, the orthogonal electric and magnetic field components are simultaneously measured at the surface, and then the apparent resistivity (in Ωm) is obtained. The penetration depth can be increased by lowering the electromagnetic wave frequency^65^. So, compared with ERT, CSAMT can provide greater investigation depth even more than 1km^17,31^. During the detection, the two pairs of electrodes and the two magnetic probes are arranged symmetrically around the measuring point.

**Figure 4:**
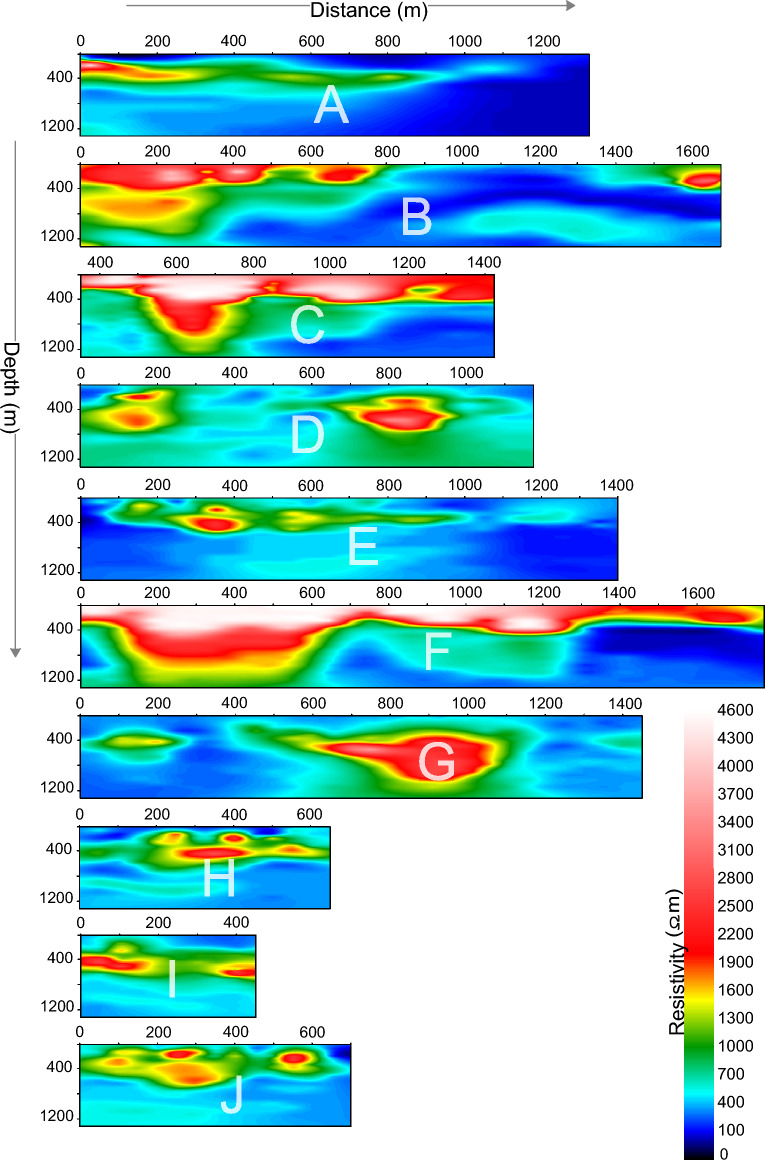
The 2D models (with resistivity increasing from dark blue to red white on a color-bar) obtained from CSAMT survey along 10 profiles A-J in JUNO site.

In this work, JUNO site was investigated by CSAMT along 10 profiles (A-J) with profile distance ranging between 450–1775 m (Fig. 1d). In this study, CSAMT provides 1500 m depth of investigation. The working parameters include 50 m point distance, 20 m pole-distance tensor measurement. According to the real-time intensity of the natural field, the high, medium and low frequency bands were used 10 to 13 times. The electric field was received by a pure titanium non-polarized electrode with a sensor; the magnetic field was received by a BF-6 high-sensitivity magnetic probe; and the detected data was acquired using an EH-4 continuous conductivity meter. The orientation of the electrode and magnetic probe is real-time positioning using the compass to ensure that the azimuth deviation is not more than 3 degrees. The horizontal ruler is used to ensure the horizontal placement of the magnetic bar. Besides, it is ensured that the electrode grounding resistance is as small as possible. In addition, we observe the data and curve from the window timely to ensure the quality of data collection. A set of Stratagem EH-4 (II) continuous conductivity imager was used. The main performance of the instrument includes frequency range: 10–92 kHz, electrode: 4 BE-26 type effective high frequency dipoles with buffer and 4 SSE stainless steel electrodes, magnetic rod probe, 2 BF-IM magnetic induction rods (10–100 Hz), working temperature: 0–50 °C, and data acquisition unit: 4 channels (2 power, 2 magnetic). There are two main softwares used in CSAMT data processing, namely CMT Pro and CSAMT-SW. CMT Pro processing includes integration of the host data, source data and reference channel data, editing of the position of the measuring point and preliminary preparation of the data. The main processing functions of CSAMT-SW software includes data conversion, elevation addition, smoothing processing, static and near-field correction, BOSTICK inversion, and Quasi-two-dimensional inversion. The 2D CSAMT models obtained from JUNO site are presented in Fig. 4.

### Estimation of geomechanical parameters

Rock mass strength is world widely assessed by geomechanical parameters^1,2,10,19,21^. RCI and RQD are the key geomechanical indices to appraise rock mass reliability in geotechnical investigations^1, 16,17,21,22^. These parameters evaluate the integrity of foundation rock. RQD is basically the percentage measure of hard rock obtained by a drill with a standard core diameter of 47.5 mm. In RQD measurements, the standard RQD is converted to one-meter sections of the borehole. Firstly, the solid core pieces over 10 cm (4 in.) obtained from a borehole are combined; afterwards the integrated hard core pieces are divided by the core-run length. The following relation is used to determine RQD^19^:

1 $$RQD = \left( {\frac{{\sum Length\;of\;Core\;Pieces > 10\;{\text{cm}}\left( {4\;{\text{in}}.} \right) }}{Total\;Core\;Run\;Length }} \right) \times 100\%$$

**Figure 5:**
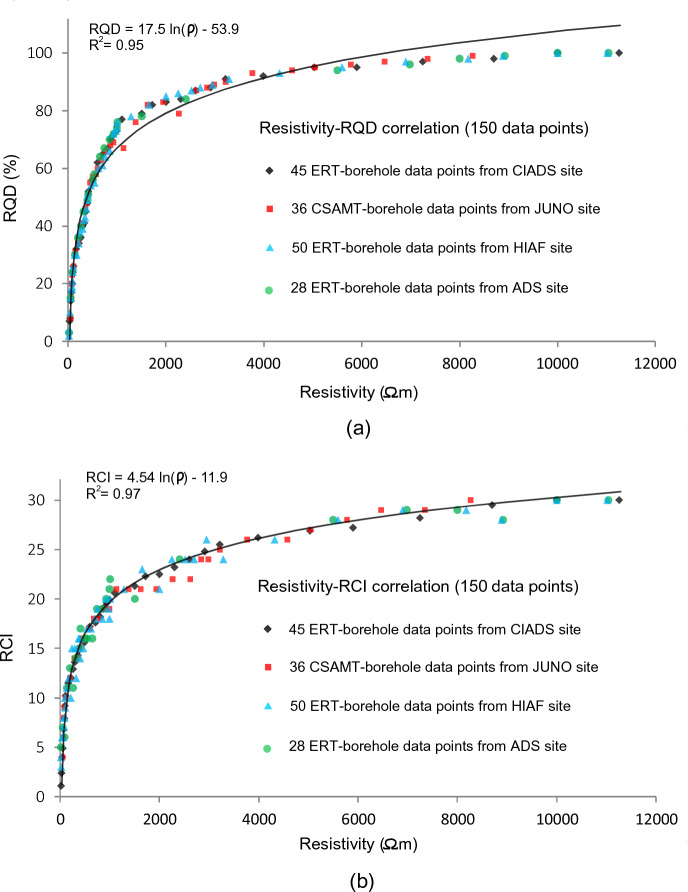
(**a**) Empirical correlation between ERT/CSAMT-based electrical resistivity and drilling-based RQD obtained from four different sites (CIADS, JUNO, HIAF and ADS), and (**b**) Empirical incorporation between ERT/CSAMT-based electrical resistivity and drilling-based RCI established from the same four sites.

Above equation is used to measure rock quality designation (RQD) in percentage (%). RCI determines continuity of the rock cores and the sampled rock mass^16^. RCI is obtained using the following equation^17^:

2 $$RCI=1\times Cr1+3\times Cr3+10\times Cr10+30\times Cr30+50\times Cr50+100\times Cr100$$

where, Cr shows the core rate of various core lengths. Cr1, Cr3, Cr10, Cr30, Cr50 and Cr100 are the proportions of core segments for different intervals of length, such as Cr1 with rock core length of 1–3 cm, Cr3 with 3–10 cm, Cr10 with 10–30 cm, Cr30 for 30–50 cm, Cr50 having 50 to 100 cm, and Cr100 greater than 100 cm. The obtained RQD and RCI depend on the drilling technique and the method of transporting the core to the laboratory. Firstly, RQD and RCI were obtained from boreholes of four different sites using Eq. (1) and (2). Afterwards, empirical correlations (Fig. 5) between borehole-based engineering parameters and ERT/CSAMT based electrical resistivity were established to estimate RQD and RCI over the entire investigated sites.

**Figure 6:**
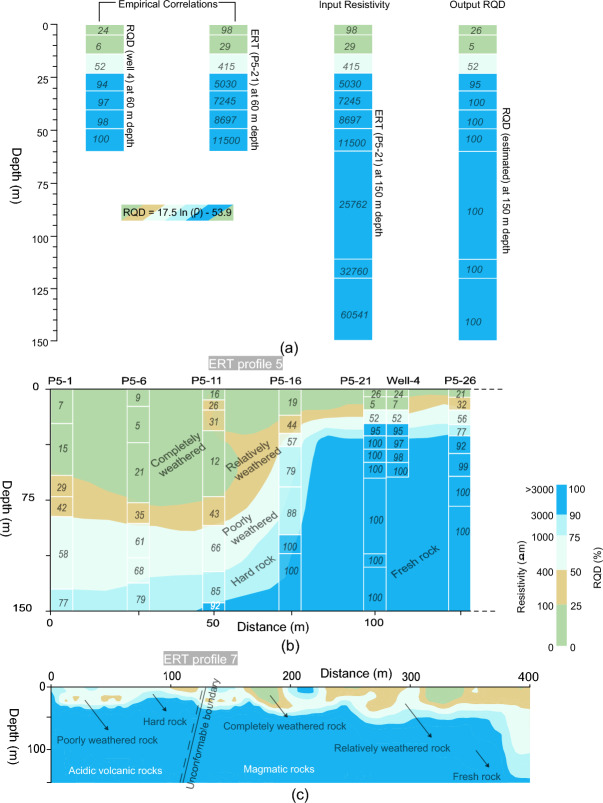
(**a**) An example of the empirical-based correlation between ERT P5-21 (21^st^ point of ERT data on profile 5 at 105 m distance) and RQD based on borehole 4, (**b**) The estimated RQD imaging along a small Sect. (0–130 m) of profile 5 based on ERT-borehole correlation, and (**c**) The lithological profile 7 obtained by ERT and RQD in CIADS site, including a description of layers/rocks and unconformable boundary between magmatic and acidic volcanic rocks.

We acquired a total of 159 RQD/RCI measurements from 25 boreholes of four sites, such as 45 measurements from CIADS site using 7 boreholes for depth 5–60 m, 36 measurements from JUNO site from 6 drill tests with depth between 10–200 m, 50 measurements from HIAF using 8 boreholes for depth ranging from 5 to 80 m, and 28 measurements from ADS site using 4 boreholes for depth between 5–40 m. Then, the obtained engineering parameters were correlated with the corresponding 159 measurements of electrical resistivity obtained from ERT/CSAMT nearby the boreholes at the same depths. Based on above borehole-resistivity correlation, the following general and adaptable equations were obtained:

3 $$RQD = 17.5 \ln \left( \rho \right) - 53.9$$

4 $$RCI = 4.54 \ln \left( \rho \right) - 11.9$$

where, ρ (in Ωm) is the inverted electrical resistivity. Equations (3) and (4) can be used to obtain RQD and RCI using resistivity of any geophysical method such as ERT and CSAMT. For resistivity below 100 Ωm, RQD and RCI range between 0–25% and 0–10 respectively; RQD 25–50% and RCI 10–15 are determined using resistivity 100–400 Ωm; for resistivity varying from 400 to 1000 Ωm, RQD 50–75% and RCI 15–20 can be obtained; similarly, RQD and RCI vary between 75–90% and 20–25 respectively for resistivity 1000–3000 Ωm; and for resistivity over 3000 Ωm, RQD 90–100% and RCI greater than 25 can be obtained. In general, the high quality rock mass is obtained for RQD 75–100% and RCI greater than 20 using resistivity above 1000 Ωm. An example of the empirical-correlation process for one data set (ERT P5-21, i.e., resistivity of ERT measurement 21 along ERT line 5; and RQD of well 4) from CIADS site is shown in Fig. 6a. Based on the correlation, the obtained 2D RQD imaging for a small Sect. (0–130 m) of ERT profile 5 from CIADS site is given in Fig. 6b. The obtained 2D RQD mapping (including detailed lithological description and interpretation) along ERT profile 7 of CIADS site is shown in Fig. 6c.

## Results

### Integration between borehole and geophysical data

Using correlation between drilling data (RQD and RCI) and geophysical data (ERT and CSAMT) of four different sites, distinct ranges of resistivity and engineering parameters divide the subsurface into various distinct layers (Table 1). The top (surface) layer of about 6 m thickness (above water table) was evaluated by the topsoil cover or overburden sediments with RQD 0–75% and RCI 0–20 for resistivity below 1000 Ωm. Second layer of completely weathered/fractured tuff rock or sandstone was evaluated with RQD 0–25% and RCI 0–10 for resistivity less than 100 Ωm. The third layer of relatively weathered/fractured tuff rock or hornstone was identified by RQD 25–50% and RCI 10–15 using resistivity 100–400 Ωm. The fourth layer of poorly weathered/fractured tuff rock or highly weathered/fractured granite was revealed with RQD 50–75% and RCI 15–20 using resistivity 400–1000 Ωm. The fifth layer of relatively integral tuff rock or poorly weathered/fractured granite was delineated by RQD 75–90% and RCI 20–25 for resistivity 1000–3000 Ωm. And the sixth layer of completely integral tuff rock or fresh granite was assessed with RQD 90–100% and RCI greater than 25 using resistivity greater than 3000 Ωm. It was observed that RQD and RCI show invariable 100% and 30 respectively for resistivity greater than 10,000 Ωm. The local subsurface geology of CIADS, HIAF and ADS sites are delineated by tuff rock as completely/highly fresh rock, relatively/poorly integral rock, poorly/partly fractured/weathered rock, relatively fractured/weathered rock and completely weathered/fractured rock unit. Whereas, the local geology of JUNO site evaluates the subsurface discrete layers as hornstone, sandstone, highly fractured or weathered granite, poorly weathered/fractured granite and completely integral or fresh granite. The subsurface layers evaluated by RQD 0–75% and RCI 0–20 using resistivity less than 1000 Ωm are assessed as the low quality rock mass, and thus such rock mass is suggested as unsuitable for construction of engineering infrastructures. Whereas, the hard/fresh geological layers via RQD from 75 to 100% and RCI greater than 20 for resistivity greater than 1000 Ωm are evaluated as rock mass of high quality and recommended as suitable for engineering construction.

**Table 1:** Classification of rock mass strength using integration between electrical resistivity (obtained from ERT and CSAMT), and engineering parameters RQD and RCI (determined from the limited borehole data).

Resistivity (Ωm)	RQD (%)	RCI	Rock mass quality	Site suitability for infrastructures
< 1000	0–75	0–20	Topsoil cover or overburden sediments (above water table)	Unsuitable
< 100	0–25	0–10	Completely weathered/fractured rock or sandstone	
100–400	25–50	10–15	Relatively weathered/fractured rock or hornstone	
400–1000	50–75	15–20	Poorly weathered/fractured rock or highly weathered/fractured granite	
1000–3000	75–90	20–25	Relatively integral rock or poorly weathered/fractured granite (hard rock)	Suitable
> 3000	90–100	> 25	Highly integral rock or fresh granite (fresh rock)	

### Rock mass quality evaluation at CIADS site

**Figure 7:**
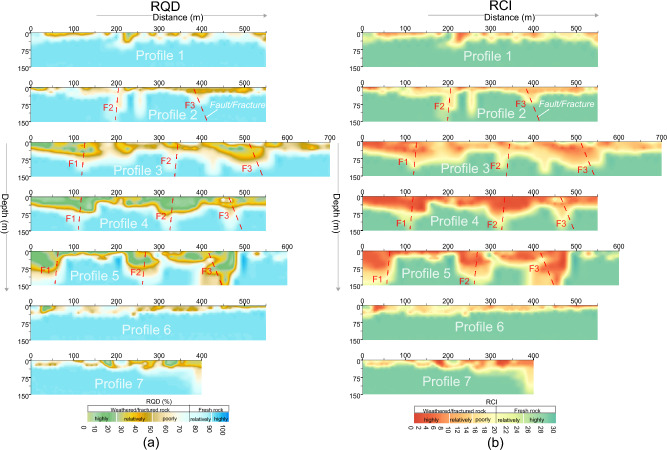
(**a**) The obtained 2D RQD models along seven geophysical profiles (1–7) in CIADS site (RQD increasing from grey green to blue on a color bar) including fractures/faults F1–F3 (dashed red lines), and (**b**) The estimated 2D RCI models along seven ERT profiles (1–7) in CIADS site (RCI increasing from red to green on a color bar) including fractures/faults F1–F3 (dotted red lines).

**Figure 8:**
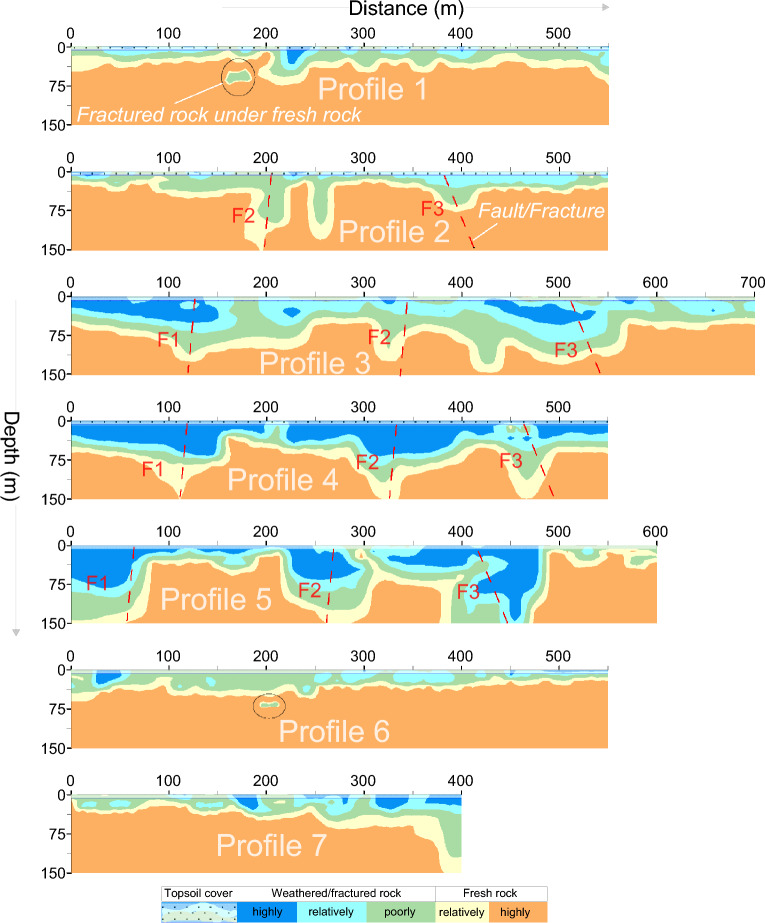
The six-layered geological models along seven ERT profiles (1–7) in CIADS site obtained from the integration of 2D RQD and RCI models, and interpreted for rock mass integrity evaluation such as topsoil cover (white area with black dots), completely weathered/fractured rock (dark blue area), relatively weathered/fractured rock (light blue region), partly fractured/weathered rock (green area), relatively integral rock (yellow region) and highly integral fresh rock (brown area) including fractures/faults F1–F3 (dashed red lines).

The obtained generalized Eqs. (3) and (4) were used to estimate engineering parameters for rock mass reliability assessment at four sites. However, detailed results of CIADS site are given in this work. In CIADS site, firstly the borehole-based RQD and RCI were used to appraise rock mass reliability for the selected seven point-locations for depth ranging from 5 to 60 m. But, the limited drilling data did not provide the in-depth estimation of rock mass potency over the complete investigated area. To reduce the gaps between scarce boreholes and the accurate subsurface models, empirical-based geophysical approach of four different sites was used to provide in-depth imaging of rock mass strength. The obtained 2D models of RQD and RCI are given in Fig. 7. The estimated RQD and RCI models evaluated the engineering rock mass at 780 points for 0–150 m depth. Based on local geological setting of CIADS site and the estimated 2D RQD and RCI models (Fig. 7), rock mass reliability was evaluated for six different layers using the specific value range of engineering parameters, such as topsoil cover or overburden sediments above water table (RQD 0–75% and RCI 0–20), completely crushed rock (RQD 0–25% and RCI 0–10), partly weathered/fractured rock (RQD 25–50% and RCI 10–15), poorly weathered/fractured rock (RQD 50–75% and RCI 15–20), relatively/poorly integral rock (RQD 75–90% and RCI 20–25) and highly integral or fresh rock (RQD 90–100% and RCI greater than 25) (Fig. 8).

**Figure 9:**
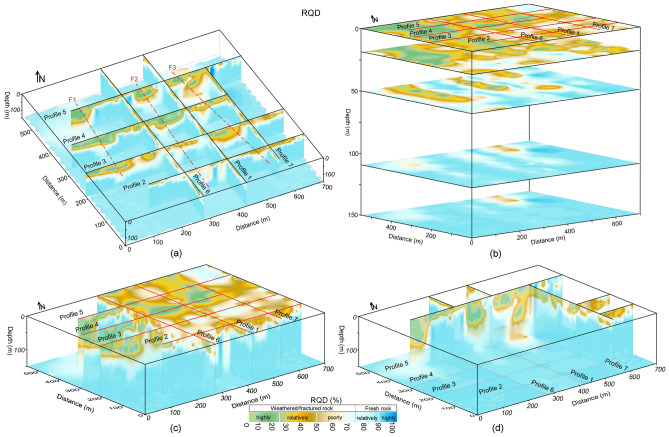
The obtained RQD imaging along seven ERT profiles (1–7) in CIADS site (increasing from grey green to blue on a color bar), including: (**a**) The incorporated 2D RQD models with RQD imaging at 150 m depth (bottom), (**b**) RQD distribution at various depths (surface or 0 m, 20 m , 50 m, 110 m and 150 m or bottom), (**c**) 3D RQD model with outer view, and (**d**) 3D RQD model with inner outlook.

**Figure 10:**
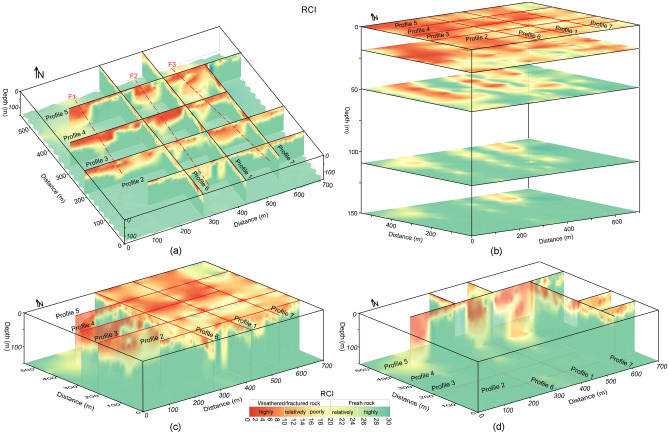
The obtained RCI imaging along seven ERT profiles (1–7) in CIADS site (increasing from red to green on a color bar), including: (**a**) The integrated 2D RCI models with RCI mapping at 150 m depth (bottom), (**b**) RCI distribution at various depths (surface or 0 m, 20 m, 50 m, 110 m and 150 m or bottom), (**c**) 3D RCI model with outer view, and (**d**) 3D RCI model with inner outlook.

The interpreted 2D models in Fig. 8 (obtained from the integration of 2D RQD and RCI models) reveal the top-surface cover with 6 m thickness throughout all profiles. Along profile 1 (Fig. 8), mostly the relatively/highly integral rock mass was delineated under depth of 35 m, including 56 m deep zone of highly/relatively weathered/fractured rock at 230 m and 545 m distance. Along surveyed line 2, engineering parameters delineated the highly fractured/weathered rock with 85 m thickness at 177–218 m, 238–267 m and 357–417 m distance, and the integral/fresh rock mass under 40 m depth at other places. Similarly RQD and RCI along profile 3 assessed several zones of highly/relatively weathered rock reaching 105 m depth from the surface, including the completely/relatively integral rock under 38 m depth at 0–68 m, 247–298 m and 566–696 distances. Along profile 4, RQD and RCI delineated the completely/relatively fresh rock under 58 m depth at 0–47 m, 157–217 m, 407–426 m and 486–548 m distance, and the highly/relatively weathered rock of 95 m thickness at other locations. RQD and RCI along profile 5 evaluated two deeper zones of highly fractured/weathered rocks exceeding the exploration depths of 150 m at 0–78 m and 207–487 m distance, including the highly/relatively integral rock under 27 m depth at other places. Along profile 6, engineering parameters assessed the completely/relatively fresh rock dominated at most of the places especially under 27 m depth. Similarly, RQD and RCI along profile 7 evaluated the highly/relatively integral rock under 25 m depth for most of the locations, and the highly fractured/weathered rock at distance of 368–398 m. Different insights of RQD imaging into the subsurface was obtained via the integrated 2D RQD models (Fig. 9a), subsurface RQD imaging at different depths (Fig. 9b), 3D RQD outer view (Fig. 9c) and 3D RQD inner view (Fig. 9d). The similar imaging of RCI with different outlooks was also obtained (Fig. 10).

**Figure 11:**
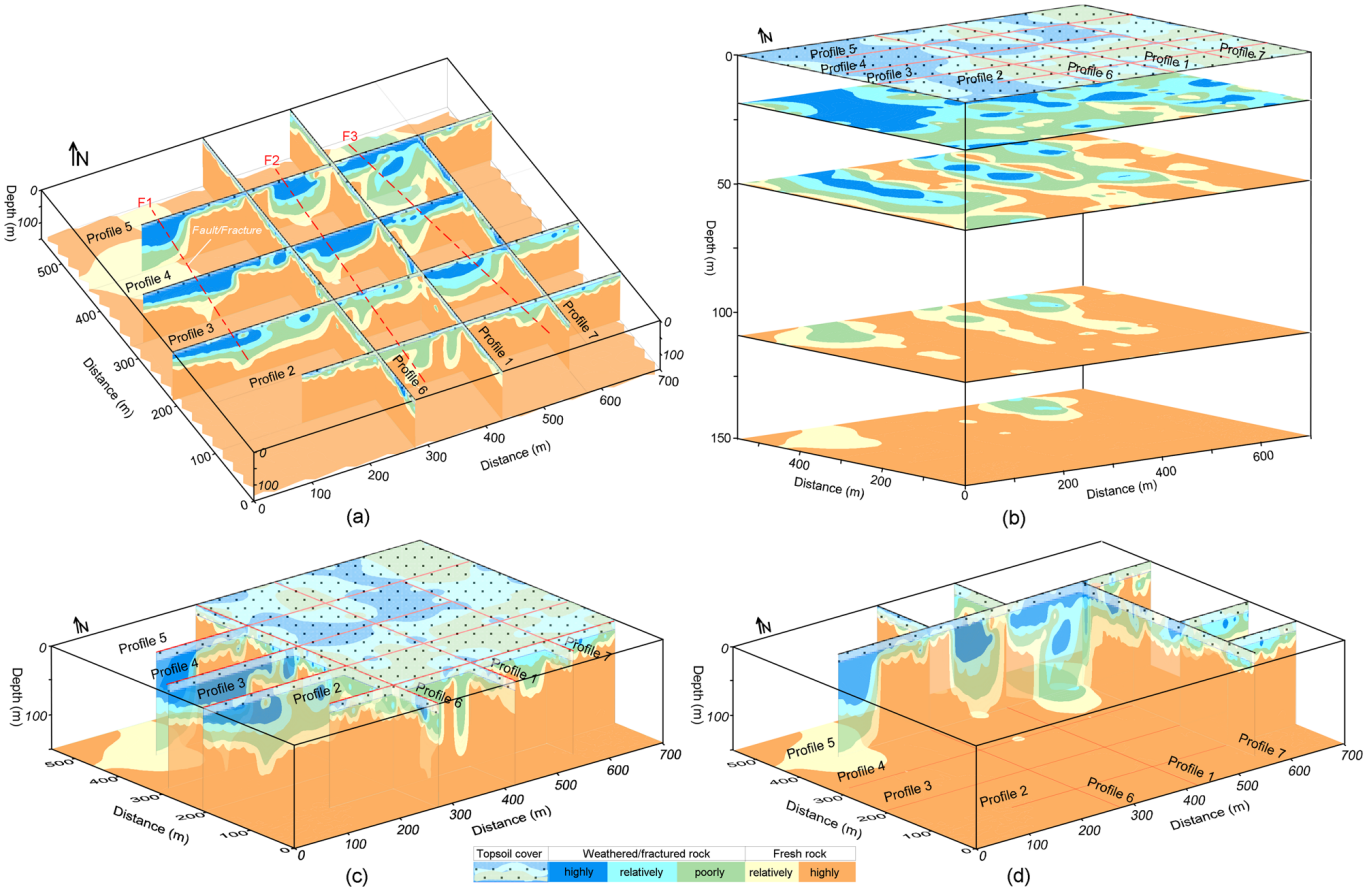
The resultant (interpreted) imaging based on RQD and RCI results for rock mass integrity assessment via topsoil cover (white area with black dots), completely weathered/fractured rock (dark blue area), relatively weathered/fractured rock (light blue region), partly weathered/fractured rock (green area), relatively integral rock (yellow region), highly integral fresh rock (brown area) and fractures/faults F1–F3 (dashed red lines), including: (**a**) The integrated 2D models of rock mass quality, (**b**) Rock mass strength mapping at various depths, (**c**) 3D appraisal of rock mass reliability with outer view, and (**d**) 3D appraisal of rock mass integrity via inner view.

Based on the detailed RQD and RCI imaging in Figs. 9 and 10, rock mass quality was comprehensively appraised by different insights into the subsurface for relatively integral fresh rock, entirely integral fresh rock, moderately fractured/weathered rock, weakly and highly weathered or fractured rock, and topsoil cover as shown in Fig. 11. The interpreted subsurface imaging (Fig. 11) reveals that profiles 1, 6 and 7 offer more proper places for engineering design, whereas profiles 3–5 suggest the unsuited places for engineering design. Generally, load-bearing capacity of engineering rock improves downward from topsoil cover to the bottom layer. It was observed that the weathered/fractured rock is dominant from surface to 50 m depth, while the subsurface at or greater than 50 m depth is mainly delineated with fresh rock. The near-surface under topsoil cover is mostly (over 90%) dominated by the highly/completely weathered rock except some zones of relatively integral rock in the southeast. At depth of about 25 m, 75% subsurface is exposed with highly/relatively weathered/fractured layer mainly in the northwest. At 50 m depth, about 55% of the subsurface is covered by the relatively/highly integral rock mostly in the east and southeast. At 110 m depth, over 90% of the subsurface is exposed by the highly/relatively integral rock except some zones of relatively crushed layer along ERT line 5 in north. Similarly at the bottom or 150 m depth, over 95% of the rock mass is the highly strong rock except one small zone of the poorly fractured rock along profile 5 in the north. Generally, the southeast is dominated by the integral/fresh rock, while the northwest is covered by fractured/weathered layer. The results reveal that a complete assessment of rock mass reliability via RQD and RCI imaging gives an acceptable subsurface model for the success of engineering design in CIADS site.

### Fractures/Faults evaluation at CIADS site

The deep and thick weathered zones were identified by fractures/faults in CIADS site. Three faults or fractures namely F1, F2 and F3 were detected in the investigated site (Figs. 7–11). All fractures/faults were delineated in northwest-southeast direction. These are the localized, intense and near-vertical fractures zones. F1 was identified along profiles 3, 4 and 5 in the west part of the study area. F1 passes through profile 3 up to 115 m depth at 125 m distance, profile 4 up to 150 m depth at 120 m distance, and profile 5 for maximum 150 m depth at 60 m distance. F2 was detected through profiles 2–5 in the middle of the investigated site with northwest-southeast orientation. F2 is identified along profile 2 up to 150 m depth at 200 m distance, profile 3 with 110 m depth at 330 m distance, profile 4 for maximum 150 m depth at 325 m distance, and profile 5 up to 150 m depth at 270 m distance. F3 was delineated along profiles 2–5 in the eastern side, mainly for 80 m depth and 390 m distance along profile 2, for 135 m depth and 520 m distance along profile 3, for 145 m depth and 470 m distance along profile 4, and for 150 m depth and 450 m distance along profile 5. The deepest and widest fractures/faults were identified along profile 5. Profile 1 and 6 do not identify any fracture/fault, and hence mostly provide suitable places for engineering constructions. However, F3 is identified along profile 7 at 400 m distance. The results suggest that the ERT-based engineering parameters can effectively identify the sub-vertical fractures/faults. One fractured zone of about 20 m thickness was identified under fresh rock along profile 1 at 170 m distance and 55–75 m depth. Another 10 m thick fractured zone under fresh rock was evaluated along profile 6 at 200 m distance and 65–75 m depth. Hence, the ERT-based RQD-RCI is capable to successfully evaluate the thin fractured zones under fresh rock at shallow depth. The places at the evaluated fractures/faults suggest inappropriate locations for infrastructure design.

### Comparison between measured and estimated geomechanical parameters

For accuracy checking of the estimated engineering parameters using general Eqs. (3 and 4), a comparison was carried out between drilling-based engineering parameters (RQD and RCI) and ERT-based engineering parameters (RQD’ and RCI’) for CIADS site (Table 2). Such comparison was carried out for seven measurements selected from a total of 159 ERT-borehole measurements. 100% matching or 0% error was obtained by the comparison of the measured and estimated engineering parameters between borehole 1 and nearby ERT data point (P2-11) at 41 m depth along profile 2. Similar comparison between the ERT and borehole 2-based data at 23 m depth along profile 3 provides 91 and 94% matching (9 and 6% error) for RQD vs RQD’ and RCI vs RCI’ respectively. Along ERT profile 4 at 14 m depth of borehole 3, 94% matching (6% error) for RQD-RQD’ and 90% matching (10% error) for RCI-RCI’ was obtained. Another strong matching (100% matching or 0% error for RQD-RQD’ and 98% matching or 2% error for RCI-RCI’) was obtained for borehole 4 at 50 m depth along profile 5. At 60 m depth of borehole 5 along profile 5, 87% matching (13% error) between RQD’ and RQD, and 83% matching (17% error) between RCI and RCI’ was observed. Along profile 6 at 5 m depth of borehole 6, 94% matching (6% error) between RQD-RQD’ and 89% matching (11% error) between RCI-RCI’ was obtained. RQD vs RQD’ with 97% matching (3% error) and RCI vs RCI’ with 92% matching (8% error) were obtained along profile 7 at 32 m depth of borehole 7. The comparison provides acceptable matching or acceptable error between the measured and obtained engineering parameters for rock mass quality evaluation.

**Table 2:** Accuracy checking of the obtained engineering parameters via matching between the borehole-based RQD/RCI and the ERT-based RQD’/RCI’ for the selected CIADS site.

ERT data (selected)				Well data				% Matching/Error (estimation of uncertainty)	
ERT data point	Resistivity (Ωm) (selected)	Estimated RQD’ (%)	Estimated RCI’	Depth (m)	Well name	Measured RQD (%)	Measured RCI	RQD’ vs RQD	RCI’ vs RCI
P2-11	11,256	100	30	41	1	100	30	100/0	100/0
P3-75	40	11	4.5	23	2	10	4.8	91/9	94/6
P4-87	262	44	13.4	14	3	47	14.8	94/6	90/10
P5-21	8697	100	29.3	50	4	100	30	100/0	98/2
P5-85	295	46	13.9	60	5	40	12.1	87/13	83/17
P6-77	871	66	18.8	5	6	62	16.8	94/6	89/11
P7-9	1505	74	21.3	32	7	72	19.6	97/3	92/8

## Discussion

Geophysical methods are progressively being used in geotechnical engineering. Several past studies suggested useful correlations between geophysical and borehole data to evaluate rock mass quality. Various engineering parameters are used in rock mass quality classification. RQD, RCI and Kv conventionally measured by boreholes are the most reliable and efficient geotechnical parameters widely used in such evaluations. In this research, we introduce an empirical-based methodology of electrical resistivity obtained from two geophysical methods ERT and CSAMT for rock mass quality evolution. Our approach provides the general and adaptable empirical correlations based on extensive geophysical and borehole data from four different sites. The obtained empirical equations are based on four different sites with different lithologies/rocks, namely topsoil cover or overburden sediments, completely/highly integral or fresh tuff rock, relatively integral tuff rock, poorly weathered/fractured tuff rock, relatively weathered/fractured tuff rock, highly/completely weathered/fractured tuff rock in CIADS, HIAF and ADS sites, whereas topsoil cover, highly/completely integral granite, relatively/poorly weathered/fractured granite, highly/completely weathered/fractured granite, hornstone and sandstone in JUNO site. Both weathered and fractured rocks show the same value of resistivity and RQD/RCI, so it is difficult to distinguish these rocks. However, the weathered rock was evaluated just under the topsoil cover at shallow depth, whereas fractured rock was assessed in the subsurface at large depths. The distinct rocks were obtained using specific value range of RQD and RCI. Generally, we divide the distinct rocks of different quality into two main categories, such as rock mass of good/high quality (RQD greater than 75% and RCI greater than 20) and rock mass of poor/low quality (RQD less than 75% and RCI less than 20) (Figs. 7–11). The obtained general equations can be used in most of the hard-rock sites to evaluate rock mass quality via RQD/RCI using resistivity data of any geophysical method. These equations can only provide the output engineering parameters for the input/given resistivity; however, the specific value ranges of RQD and RCI are interpreted based on the local lithology and rock type. For example, in this research, the lowest quality of rock mass is appraised via highly or completely fractured/weathered tuff-rock in CIADS, HIAF and ADS sites but sandstone in JUNO site using the same value range of RQD 0–25% and RCI 0–10 for resistivity less than 100 Ωm. Hence the specific name of low or high quality of rock mass is based on the local geological information; however the general interpretation/evaluation of high/low integrity rock mass can be obtained for any site using the established general/adaptable equations.

The empirical correlation was performed between the in-situ resistivity measurements and RQD/RCI measurements obtained from rock cores of boreholes. Alternatively, one empirical correlation can be established between the laboratory-based resistivity of rock cores and the in-situ resistivity, which can be used to obtain laboratory-based (rock cores) resistivity along all in-situ (ERT/CSAMT) resistivity data then another empirical correlation between laboratory-based resistivity and drilling-based RQD/RCI can be performed to determine engineering parameters using the estimated laboratory-based resistivity along all in-situ geophysical profiles. By this way, the estimated engineering parameters obtained via two different empirical correlations would provide more error (at least 2 times more than shown in Table 2). This correlation was performed for 159 resistivity-borehole data sets with the lowest and highest limit value such as 27–11,650 Ωm for resistivity, 4–100% for RQD and 2–30 for RCI. Resistivity ranges between 25 and 90,000 Ωm along all geophysical profiles, RQD with 100% and RCI with 30 remain constant for any resistivity above 10,000 Ωm. Hence, even though this correlation was carried out for depth 5–80 m in the ERT survey sites and 10–200 m in the CSAMT survey site but it provides the complete range of engineering parameters (0–100% for RQD and 0–30 for RCI) for all types of rocks covering the entire depths of geophysical profiles over the whole sites. That’s why the established correlation (Eqs. 3 and 4) provides strong matching or less error between the obtained and measured engineering parameters (Table 2). An assessment of uncertainty in the estimation/prediction of geotechnical parameters (using geophysical data) from CIADS site was performed by % matching/error in Table 2. The comparison (in Table 2) between the predicted geotechnical parameters (obtained from geophysical data) and the measured geotechnical parameters (obtained from borehole data) show strong matching (more than 90%) and low error (less than 10%) for most of the measurements for the selected data sets. Thus, the predicted geotechnical models have good (acceptable) accuracy. The accuracy of the predicted geotechnical models depend on several factors, including the accuracy of geophysical models and borehole-based geotechnical parameters, and strong empirical correlation between the geophysical and geotechnical parameters (i.e., R^2^ > 0.9). From borehole data, we obtain geotechnical parameters with the known geology and then we integrate borehole data (measured geotechnical parameters and known geology) with geophysical data via empirical correlation to get the predicted geotechnical parameters.

The suitable geophysical method is selected based on objectives and requirements of the investigation. However, geophysical methods also have limitations. A geophysical method alone cannot give accurate results until the known geological information (from borehole data) is integrated. Besides, use of single geophysical method may provide ambiguity in the model results which can be removed by integrating two or more geophysical methods. The 2D/3D electrical resistivity methods can be the most practical cost-effective compromise between the low survey cost and acceptable accurate results. However, through acquisition, processing and interpretation, the uncertainties may affect the accuracy of resistivity model results. Different environmental/geological settings provide variety of data sets. However, the inversion technique with different settings of inversion parameters is capable to reduce the inverted model uncertainty and provide the results closer to the known geology. The subsurface is assumed as 2D when the resistivity measurements are acquired along a line. Such hypothesis is only applicable if the resistivity surveys are performed across strikes of the elongated structures. However, the 2D inverted model may cause uncertainty in the lower sections if the subsurface resistivity varies significantly with directions perpendicular to the surveyed lines. Such ambiguity caused by 3D structures can be removed by performing data inversion of a full 3D resistivity survey. Thus, if the assumption of 2D geology is wrong then the 2D resistivity survey will give the ambiguous results even with reliable inversion algorithms, excellent data quality and dense data coverage. The predicted/obtained 2D models of geotechnical parameters (Figs. 7 and 8) show high resolution at shallow depth, but resolution decreases downward with the depth. The predicted 3D models (Figs. 9–11) show more reliability mainly near the geophysical profiles at shallow depth; however, the resolution decreases with increasing the distance from surveyed lines as well as with increasing the depth. The resolution of electrical resistivity methods is poorer than seismic or GPR methods at large depths. Resistivity resolution decreases exponentially with depths, and it is ambiguous to evaluate a subsurface structure of 1 m size below 10 m depth. In the inversion of resistivity data, the problem of non-uniqueness is well known not only in 1D resistivity modeling but also in 2D/3D models. This problem occurs when the same response is produced from more than one model which can agree with the obtained resistivity within the limits of data precision. Such uncertainty can be resolved by integrating the inversion subroutine with the known nature of subsurface. The data noise causes uncertainty in the inversion models, which can be removed by strong electrode-ground contact and elimination of bad data (with unusual low or high value) manually or in the inversion procedure. The resistivity inversion technique tries to minimize the difference between the measured and calculated resistivity. However, an inversion model with 5% RMS error for one data set may not provide the same model with 5% RMS error for another data set even though both belong to the same place. The success of resistivity surveys depends on current penetration into the ground. If surface layers have very low or high resistivity, current can be trapped in the top layer and thus enough current may not flow through the subsurface which may provide ambiguous results from the lower layers. Given the subsurface heterogeneities, resistivity varies from one area to another even within the same area which may cause uncertainty in the predicted geotechnical models. This research, compared to the previous work, removes such ambiguity in the predicted models by using extensive geophysical-borehole data of four different sites via integration of two geophysical methods. The proposed approach cannot be used without the integration of drilling data. However, it can reduce lots of boreholes to obtain geotechnical parameters over the entire investigated site. The obtained equations can be used in the sites where boreholes are not accessible to obtain geotechnical parameters.

It was found that the obtained RQD and RCI models show good matching with each other for the appraisal of rock mass reliability. The abrupt changes at various layer-interfaces can affect RQD, since RQD is obtained by the joint borehole lengths with the small core units. However, compared with RQD, RCI is estimated by multiple scales and thus cannot be affected by layer fractures. RCI provides more accuracy than RQD for low values suggesting low-quality rock mass, while RQD is more accurate for high-quality rock mass with high value. RCI defines the underground mass structure better than RQD. The highest integrity of rock mass was evaluated by RQD 100% and RCI 30 for resistivity greater than 10,000 Ωm. But, in comparison with RQD and RCI which remain constant at 100% and 30 respectively for resistivity over 10,000 Ωm, ERT and CSAMT are capable to further estimate the integrity of engineering rock and show increase in integrity of rock mass even over 10,000 Ωm. The highest values of resistivity are related to the highest integrity of rock mass. The construction design in the sites was modified based on the obtained RQD and RCI models. Furthermore, the borehole-based engineering parameters show nearly the constant imaging of geological layers and may not delineate fractures/faults, while the ERT/CSAMT-based engineering parameters provide accurate imaging of subsurface layers including perfect identification of fractures/faults (F1, F2 and F3). Therefore, the inadequate drilling tests leave many ambiguities in the evaluation of geological models. However, empirical-based geophysical approach of ERT and CSAMT can bridge the gap between the accurate subsurface models and insufficient boreholes.

## Conclusions

This research provides the applications of non-invasive geophysical methods such as ERT and CSAMT in rock engineering for a comprehensive valuation of rock mass potency. The empirical-based approach of ERT and CSAMT offers the most important engineering parameters RQD and RCI for the appraisal of rock mass reliability over large areas where even no drilling data exists. Conventionally, engineering parameters are measured using boreholes. However, borehole approaches are expensive and have several limitations. Therefore, alternatively, our approach reduces an extensive number of boreholes, and provides far better and thorough evolution of rock mass quality compared with the past geotechnical tests. In this work, based on geophysical data (resistivity obtained from several ERT and CSAMT profiles) and borehole data (RQD and RCI obtained from limited drilling tests) of four different sites, we obtained general and adaptable equations to estimate RQD and RCI for distinct layers of high and low quality of engineering rock. The established general/adaptable formulas were used to determine RQD and RCI in the selected CIADS site for a comprehensive estimation of rock mass integrity with distinct layers, namely topsoil cover or overburden sediments (above water table) with RQD 0–75% and RCI 0–20 using resistivity less than 1000 Ωm, completely or highly weathered/fractured rock with RQD 0–25% and RCI 0–10 using resistivity less than 100 Ωm, relatively weathered/fractured rock for RQD 25–50% and RCI 10–15 using resistivity 100–400 Ωm, poorly weathered/fractured rock having RQD 50–75% and RCI 15–20 using resistivity 400–1000 Ωm, relatively integral rock with RQD 75–90% and RCI 20–25 using resistivity 1000–3000 Ωm, and completely integral rock for RQD 90–100% and RCI greater than 25 with resistivity > 3000 Ωm. The results suggest that low resistivity corresponds to low RQD/RCI; similarly, high resistivity shows high RQD/RCI. However, specific range of resistivity-RQD/RCI for various rock mass units is relative but not absolute and may change from one site to another. The obtained empirical equations of RQD and RCI can be used in other areas; but the local known geology is suggested to be integrated with the predicted geotechnical models in order to accurately interpret the local rock mass units. Three localized fractures/faults (F1, F2 and F3) were detected in northwest-southeast orientation. The results suggest that the potency of engineering rock increases via increment in RQD and RCI values. The weathered or fractured rock units and fractures/faults are considered as the poor integrity of rock unit, and are recommended as improper for engineering construction. The unweathered rock is suggested as high quality rock unit with suitability for engineering construction. The results of RCI and RQD models match with each other and suggest high correlation with the local geology. The predicted geotechnical models were confirmed/validated by the drilling data and the subsurface geological conditions of the investigated site. The predicted models reveal that this approach can be good substitute of the costly boreholes to attain far better mapping of geological models compared with the conventional geotechnical approaches. Therefore, the use of geophysical methods can be highly successful in geotechnical engineering to provide a thorough assessment of rock mass quality, and bridge the gap between the true geological models and scarce borehole data.

## Data Availability

Data available on request from the corresponding author.

## Code availability

Software application or custom code support the published claims and comply with field standard.
